# Programmed cell death during neuronal development: the sympathetic neuron model

**DOI:** 10.1038/cdd.2014.47

**Published:** 2014-04-25

**Authors:** M Kristiansen, J Ham

**Affiliations:** 1 Molecular Haematology and Cancer Biology Unit, Institute of Child Health, University College London, 30 Guilford Street, London WC1N 1EH, UK

## Abstract

Developing sympathetic neurons of the superior cervical ganglion are one of the best studied models of neuronal apoptosis. These cells require nerve growth factor (NGF) for survival at the time that they innervate their final target tissues during late embryonic and early postnatal development. In the absence of NGF, developing sympathetic neurons die by apoptosis in a transcription-dependent manner. Molecular studies of sympathetic neuron apoptosis began in the 1980s. We now know that NGF withdrawal activates the mitochondrial (intrinsic) pathway of apoptosis in sympathetic neurons cultured *in vitro*, and the roles of caspases, Bcl-2 (B-cell CLL/lymphoma 2) family proteins and XIAP (X-linked inhibitor of apoptosis protein) have been extensively studied. Importantly, a considerable amount has also been learned about the intracellular signalling pathways and transcription factors that regulate programmed cell death in sympathetic neurons. In this article, we review the key papers published in the past few years, covering all aspects of apoptosis regulation in sympathetic neurons and focusing, in particular, on how signalling pathways and transcription factors regulate the cell death programme. We make some comparisons with other models of neuronal apoptosis and describe possible future directions for the field.

## Facts

- Developing NGF-dependent sympathetic neurons are a very well characterised model of neuronal apoptosis.
- NGF withdrawal-induced death *in vitro* requires *de novo* gene expression, as does the death of other kinds of primary neuron, including developing cerebellar granule neurons (CGNs), motor neurons and cortical neurons.
- NGF deprivation activates the mitochondrial pathway of apoptosis, and BH3-only proteins and Bax (Bcl-2-associated x protein) are required for mitochondrial outer membrane permeabilisation (MOMP) and cytochrome *c* release.
- The binding of NGF to TrkA activates the PI3K-Akt (phosphatidylinositol 3-kinase-Akt) and Raf-MEK-ERK (Raf-MAPK/extracellular signal-regulated kinase kinase-extracellular signal-regulated kinase) signalling pathways, which promote the growth and survival of sympathetic neurons.
- NGF deprivation decreases the activity of the PI3K-Akt and Raf-MEK-ERK survival pathways, but increases the activity of the MLK-JNK-c-Jun (mixed lineage kinase-c-Jun N-terminal kinase-Jun proto-oncogene) pathway, which is required for the increased expression of BH3-only proteins and for mitochondrial cytochrome *c* release.

## Open Questions

- How is the PI3K-Akt pathway inactivated after NGF withdrawal and how is the JNK pathway activated?
- How exactly do TrkA and p75NTR (p75 neurotrophin receptor) regulate NGF withdrawal-induced death?
- How do the new NGF-regulated genes identified by gene microarray analysis contribute to the control of sympathetic neuron death and survival?
- How do the core cell death proteins in sympathetic neurons function in axon degeneration induced by local NGF deprivation?
- How similar are the mechanisms of cell death in sympathetic neurons and developing central nervous system neurons, such as CGNs or cortical neurons?

Apoptosis occurs extensively during the normal development of the mammalian nervous system and has been observed in populations of developing neural precursor cells, differentiated postmitotic neurons and glial cells.^1, 2, 3^ These cell deaths are important for establishing neuronal and glial populations of the correct size. In the case of the developing peripheral nervous system (PNS), neuronal apoptosis has been shown to be important for matching the number of innervating neurons to the size of the final targets that they innervate. Sympathetic neurons of the superior cervical ganglion (SCG) have been extensively studied as a model of naturally occurring neuronal death in the PNS. During mammalian development, one-third of these cells normally die by apoptosis during the first 2 weeks after birth.^4^ At this time, sympathetic neurons require nerve growth factor (NGF), synthesised by their target tissues, for survival.^5^ NGF is produced in limiting amounts by the targets innervated by SCG neurons, and binds to its specific tyrosine kinase receptor, TrkA, on the surface of the innervating axons.^5^ The NGF–TrkA complex is then retrogradely transported to the sympathetic neuron cell bodies and promotes neuronal growth. Importantly, the binding of NGF to TrkA also inhibits neuronal apoptosis. Levi-Montalcini and Booker^6, 7^ showed that injection of a neutralising anti-NGF antiserum into early postnatal rats or mice greatly reduced the number of SCG neurons, whereas injection of purified NGF increased their number.^6, 7^ In agreement with these classic studies, targeted knockout of the *TrkA* or *Ngf* genes in mice also reduces the number of SCG neurons by increasing the amount of neuronal death that occurs.^5, 8, 9^

## Basic Features of Sympathetic Neuron Death *In Vitro*

**Figure 1:**
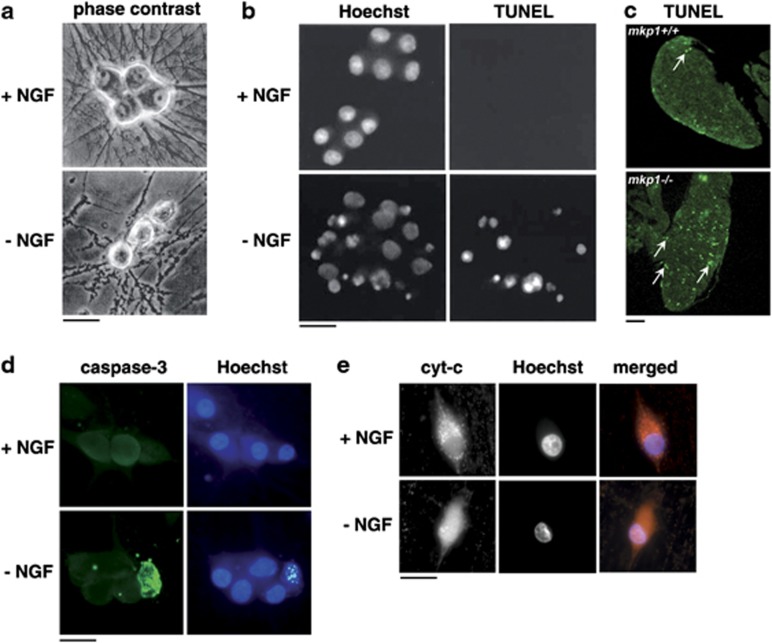
Morphological and biochemical changes that occur in sympathetic neurons undergoing programmed cell death *in vivo* or following NGF withdrawal *in vitro*. (**a**) Morphology of sympathetic neurons isolated from one-day-old rats and cultured for 6 days *in vitro* and then in the presence and absence of NGF for 48 h. Bar, 25 *μ*m. (**b**) Apoptotic chromatin condensation and DNA fragmentation in cultured sympathetic neurons visualised by Hoechst 33342 staining and TUNEL analysis. The neurons were isolated from one-day-old rats, cultured for 6 days *in vitro* and then in the presence and absence of NGF for 24 h. Bar, 25 *μ*m. (**c**) TUNEL analysis of apoptosis in the superior cervical ganglia of one-day-old wild-type and *mkp1−/−* mice. The *mkp1−/−* knockout mutation significantly increases the number of TUNEL-positive cells per ganglion.^14^ Scale bar, 100 *μ*m. (**d**) NGF withdrawal activates caspase-3 in sympathetic neurons. Neurons were cultured in the presence or absence of NGF for 48 h. The cleaved form of caspase-3 and nuclear morphology were visualised by staining the neurons with an anti-active caspase-3 antibody and Hoechst 33342. Bar, 25 *μ*m. (**e**) Distribution of cytochrome *c* in normal and apoptotic sympathetic neurons visualised by immunocytochemistry with an anti-cytochrome *c* antibody. In the presence of NGF, cytochrome *c* immunoreactivity is excluded from the nuclear space and has a punctate pattern. In the absence of NGF, a fainter, diffuse staining pattern that occurs throughout the whole cell is observed. Bar, 25 *μ*m

Developing sympathetic neurons can be isolated from the SCGs of early postnatal rats or mice, separated from other cell types and cultured *in vitro* for extended periods in medium containing NGF. When deprived of NGF, sympathetic neurons die over a period of 48–72 h and this death has the classic hallmarks of apoptosis^10, 11, 12^ (Figure 1). After NGF withdrawal, sympathetic neurons become atrophied and their neurites fragment (Figure 1a). There is also a decrease in glucose uptake and a fall in the overall rates of protein synthesis and gene transcription.^10, 11^ The nuclei of NGF-deprived neurons become pyknotic (Figure 1b) and the chromosomal DNA fragments. This can be detected as a nucleosomal DNA ladder on a gel^12^ and visualised at the single neuron level by terminal deoxynucleotidyl transferase dUTP nick-end labelling (TUNEL) analysis (Figure 1b). *In vivo*, TUNEL-positive cells can be detected in the SCGs of mice during the period of developmental neuronal death, and the number of TUNEL-positive cells per SCG is altered by mutations that change the rate of NGF withdrawal-induced death *in vitro*^13, 14^ (Figure 1c). Importantly, the NGF withdrawal-induced death of sympathetic neurons *in vitro* is strongly delayed by inhibitors of transcription or protein synthesis, suggesting that *de novo* gene expression is required for the activation of the cell death programme in these cells.^10, 12^ This is also true for cultured rat or mouse CGNs deprived of survival signals,^15, 16^ chick motor neurons deprived of trophic support^17^ or cortical neuron apoptosis,^18^ suggesting that this is often a feature of developmental neuronal death.

**Figure 2:**
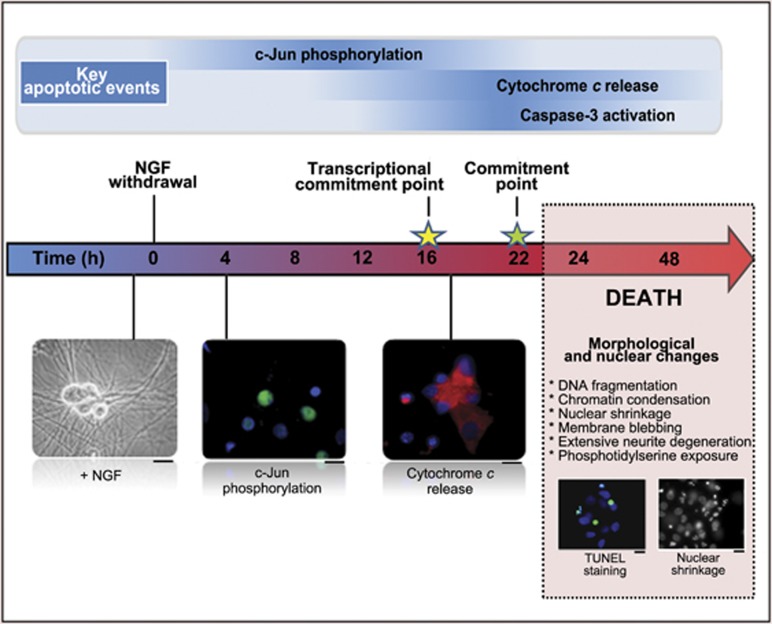
Key events following NGF withdrawal in sympathetic neurons. Sympathetic neurons undergo death 24–48 h after NGF withdrawal. The N-terminal phosphorylation of c-Jun, the release of cytochrome *c* from the mitochondria and the activation of caspase-3 are key biochemical changes seen in NGF-deprived sympathetic neurons. By ∼16 h after NGF withdrawal, only 50% of the neurons can be rescued by the addition of inhibitors of transcription or protein synthesis (the transcriptional commitment point) and by ∼22 h after NGF withdrawal only 50% of the neurons can be rescued by the readdition of NGF (the commitment point for NGF withdrawal-induced death). By 48 h, almost all of the neurons have undergone apoptosis and the nuclear and morphological changes typical of apoptosis are apparent. Images represent snapshots of NGF-deprived sympathetic neurons at the timepoints shown. Scale bars, 20 *μ*m

The timecourse of key events during NGF withdrawal-induced death has been carefully documented^10, 11^ (Figure 2). By ∼22 h after NGF withdrawal, only 50% of the neurons can be rescued by the readdition of NGF and this was defined as the commitment point for NGF withdrawal-induced death. The transcriptional commitment point is ∼16 h after NGF withdrawal and this is the time at which only 50% of the neurons can be rescued by the addition of inhibitors of transcription or protein synthesis (Figure 2).

## NGF Withdrawal Activates the Mitochondrial (Intrinsic) Pathway in Sympathetic Neurons

**Figure 3:**
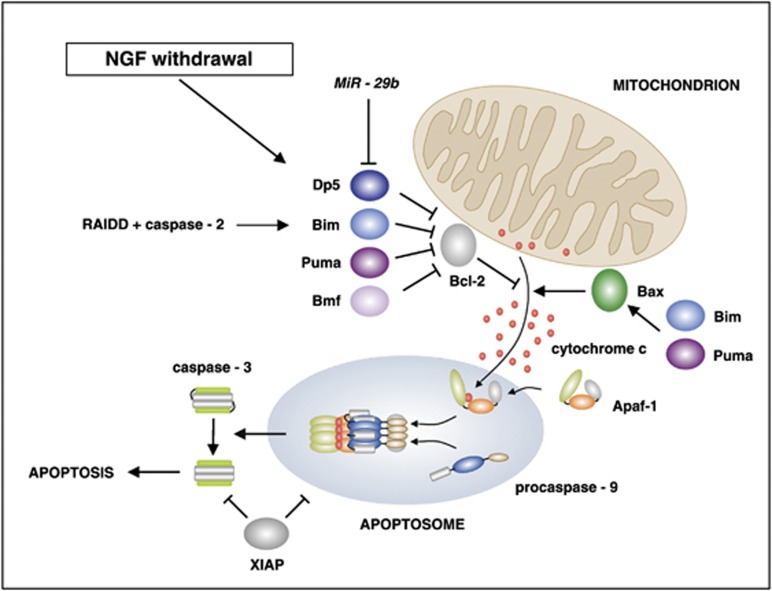
NGF withdrawal activates the mitochondrial (intrinsic) pathway of apoptosis in sympathetic neurons. After NGF withdrawal, the expression of the BH3-only proteins Dp5, Bim, Puma and Bmf increases.^35, 53, 54^ These promote MOMP by binding to and antagonising the antiapoptotic Bcl-2 and Bcl-x_L_ proteins or by directly activating Bax. The multidomain proapoptotic Bax protein is activated by NGF withdrawal and essential for MOMP and mitochondrial cytochrome *c* release. Cytosolic cytochrome *c* interacts with Apaf-1 and procaspase-9 to form the apoptosome complex, which then cleaves and activates the effector caspase, caspase-3. Bim, Puma, Bax, cytochrome *c*, Apaf-1, caspase-9 and caspase-3 have all been shown to be essential for normal NGF withdrawal-induced death in experiments using sympathetic neurons isolated from specific knockout mice or, in the case of cytochrome *c*, a neutralising anti-cytochrome *c* antibody.^23, 24, 25, 37, 41, 48, 56, 57^ RAIDD and caspase-2 may function upstream of the mitochondrial pathway in sympathetic neurons and promote cell death by increasing the expression of the BH3-only protein Bim.^32^ In the presence of NGF, XIAP inhibits caspases in sympathetic neurons, but after NGF withdrawal, the level of the XIAP protein significantly decreases,^27^ allowing the intrinsic pathway to activate caspase-3 and induce cell death. During the later postnatal development of sympathetic neurons, the level of the *MiR-29b* microRNA increases (from P13 onwards) and this inhibits expression of the BH3-only proteins and contributes to the resistance of late postnatal (P28) sympathetic neurons to NGF deprivation-induced death^54, 118^

The NGF withdrawal-induced death of sympathetic neurons requires caspase activity. Activated caspase-3 can be detected after NGF withdrawal *in vitro* (Figure 1d) and in SCGs *in vivo* during the period of naturally occurring neuronal death. Caspase inhibitors, such as BAF (boc-aspartyl-(OMe)-fluoromethyl-ketone), zVAD-fmk (carbobenzoxy-valyl-alanyl-aspartyl-[O-methyl]- fluoromethylketone) or the baculovirus p35 protein protect sympathetic neurons against cell death after NGF deprivation.^19, 20, 21, 22^ NGF withdrawal activates the mitochondrial (intrinsic) pathway of caspase activation (Figure 3). Cytochrome *c* is released from the mitochondria of NGF-deprived sympathetic neurons and this can be visualised by cell fractionation and immunoblotting^22^ or by immunocytochemistry using an anti-cytochrome *c* antibody^23, 24^ (Figure 1e). Importantly, microinjection of a neutralising anti-cytochrome *c* antibody inhibits NGF withdrawal-induced death, suggesting that the released mitochondrial cytochrome *c* is functionally important.^24^ Deletion of the *Apaf-1* (apoptotic protease-activating factor-1), *caspase-9* or *caspase-3* genes in mice prevents apoptosis after NGF deprivation, and allows the sympathetic neurons to recover and survive long-term following readdition of NGF.^25^ The readdition of NGF to NGF-deprived sympathetic neurons in which cytochrome *c* has been released leads to the refilling of the mitochondria with cytochrome *c*.^22, 26^ Sympathetic neurons are one of the few cell types in which this occurs, making this a useful model for understanding the mitochondrial death commitment point in general. Overall, these results are consistent with a model in which mitochondrial cytochrome *c* release promotes the formation of the apoptosome, which leads to the activation of caspase-9, which then cleaves and activates caspase-3 (Figure 3). Caspase-3 is critical for NGF withdrawal-induced death because early postnatal sympathetic neurons do not express the related executioner caspase, caspase-7.^25^ Another interesting feature of the sympathetic neuron model is that microinjection of purified, functional cytochrome *c* into sympathetic neurons cultured in the presence of NGF does not induce apoptosis.^23, 24^ Sympathetic neurons only become competent to die in response to cytochrome *c* injection when they have been deprived of NGF for several hours.^23^ Subsequent experiments demonstrated that after NGF withdrawal, the endogenous caspase inhibitor, X-linked inhibitor of apoptosis protein (XIAP), substantially decreases in level and that this is the molecule that protects sympathetic neurons maintained in NGF-containing medium against apoptosis induced by injection of cytochrome *c*^27^ (Figure 3).

The role of another initiator caspase –caspase-2 – has also been studied in sympathetic neurons. Caspase-2 is activated after NGF withdrawal and this activation requires the adaptor protein RAIDD (RIP-associated ICH-1/CAD-3 homologous protein with a death domain).^28^ Knockout of the *caspase-2* gene in mice does not delay NGF withdrawal-induced death *in vitro*.^29, 30^ However, caspase-9 levels are increased in the brain and in sympathetic neurons isolated from *capase-2* knockout mice, compared with the same tissues from wild-type mice, suggesting that compensatory changes in caspase-9 expression have occurred.^31^ Knockdown of caspase-2 or RAIDD in wild-type neurons using siRNAs does reduce the rate of NGF withdrawal-induced death *in vitro*.^28, 32^ Interestingly, recent results suggest that caspase-2 may function upstream of the mitochondrial pathway in sympathetic neurons and promote cell death by increasing the phosphorylation of c-Jun and the expression of the BH3-only protein Bim (Bcl-2-interacting mediator of cell death)^32^ (Figure 3).

Bcl-2 (B-cell CLL/lymphoma 2) family proteins have a key role in regulating the release of mitochondrial cytochrome *c* after NGF withdrawal^33, 34, 35^ (Figure 3). Overexpression of Bcl-2 protects sympathetic neurons against NGF withdrawal-induced death^36^ and inhibits mitochondrial cytochrome *c* release.^37^ Conversely, sympathetic neurons from *bcl-2−/−* knockout mice die more rapidly after NGF withdrawal, indicating that the endogenous Bcl-2 protein promotes sympathetic neuron survival.^38^ The multidomain proapoptotic proteins Bax and Bak (Bcl-2-antagonist/killer) are critical for MOMP, and in many cell types, both proteins are functionally important.^39^ However, in the case of sympathetic neurons, only Bax is required for mitochondrial cytochrome *c* release and NGF withdrawal-induced death. Overexpression of Bax in sympathetic neurons is sufficient to induce cytochrome *c* release and apoptosis in the presence of NGF.^37, 40^ Importantly, sympathetic neurons isolated from *bax−/−* knockout mice are strongly protected against NGF withdrawal-induced death *in vitro* and will survive for extended periods in the absence of NGF, although the neurons still become atrophied.^41^ In addition, the number of sympathetic neurons isolated from the SCGs of postnatal day 1 (P1) *bax−/−* mice is increased by 2.5-fold compared with wild-type mice.^41^ On the other hand, inactivation of the *bak* gene in mice has no effect on the rate of NGF withdrawal-induced death.^42^ Interestingly, sympathetic neurons express the *N-Bak* transcript, which is a neuron-specific splice variant of the *Bak* mRNA.^43^ The variant Bak protein encoded by this transcript only retains the BH3 domain and lacks the other BH domains. However, the N-Bak protein is not expressed in sympathetic neurons and this is due to translational repression mediated by sequences in the 5′- and 3′-UTRs of the *N-Bak* mRNA.^44, 45^

How does NGF withdrawal regulate the activity of Bax and Bcl-2 in sympathetic neurons? Bax translocates to the mitochondria after NGF withdrawal and both this and mitochondrial cytochrome *c* release are inhibited by cycloheximide, suggesting that *de novo* protein synthesis is required for Bax translocation and MOMP.^24, 46^ The expression of four different BH3-only members of the Bcl-2 family – Dp5 (neuronal death protein Dp5), Bim, Puma (p53 upregulated modulator of apoptosis) and Bmf (Bcl-2 modifying factor) – increases after NGF withdrawal^37, 47, 48, 49, 50, 51, 52, 53^ (Figure 3). Furthermore, the Bim_EL_ (the largest isoform of Bim), Puma and Bmf proteins clearly increase in level after NGF deprivation and this starts before the transcriptional commitment point.^37, 48, 50, 51, 54^ Sympathetic neurons have been isolated from the SCGs of knockout mice specific for the different BH3-only protein genes and the effect of each mutation on NGF withdrawal-induced death has been studied. Knockout of *bad* (Bcl-2-associated agonist of cell death) or *bid* (BH3 interacting domain death agonist) has no effect on the rate of cell death after NGF deprivation^42^ and the inactivation of *dp5* only has a minor effect on NGF withdrawal-induced death.^55, 56^ The role of the *bmf* gene has not been studied yet. However, knockout of either *bim* or *puma* very significantly delays cell death after NGF withdrawal, suggesting that Bim_EL_ and Puma have important roles.^37, 48, 56, 57^ In both cases, the protection against cell death is partial and this may be because Puma can partially compensate for the loss of Bim and *vice versa*. This hypothesis could be tested by culturing sympathetic neurons from *bim−/− puma−/−* double-knockout mice. This type of experiment has been carried out with mouse CGNs: knockout of *bim* or *puma* or *bid* only partially protects CGNs against apoptosis induced by survival signal withdrawal (extracellular KCl deprivation) but *bim−/− puma−/− bid−/−* triple knockout CGNs are highly resistant to apoptosis induced by KCl deprivation.^58^ The role of *bim* and *puma* has not yet been studied in developing SCG neurons *in vivo*, but knockout of *bim* has been shown to significantly reduce the number of TUNEL-positive dorsal root ganglion neurons during embryonic development, at E14.5 or E15.5.^48, 59^ In the case of sympathetic neurons *in vivo*, it would be interesting to compare the number of TUNEL-positive neurons per SCG and the total number of SCG neurons in *bim −/− puma −/−* double-knockout mice, *bim −/−* mice, *puma −/−* mice and wild-type mice, at the time of naturally occurring neuronal death.

In cultured sympathetic neurons, Bim_EL_ has been shown to be present at the mitochondria after NGF withdrawal^48^ and overexpression of Bim_EL_ in the presence of NGF is sufficient to trigger mitochondrial cytochrome *c* release and apoptosis.^37^ These results suggest a model in which Dp5, Bim, Puma and Bmf rapidly increase in level after NGF withdrawal and promote MOMP in sympathetic neurons, with Bim and Puma having major roles, by binding to antiapoptotic Bcl-2 family proteins, such as Bcl-2, and thereby preventing them from inhibiting Bax-dependent cytochrome *c* release (Figure 3). In addition, Bim_EL_ and Puma may also directly bind to and activate Bax, as suggested by work on Bax in other systems.^39, 60, 61, 62^

## Changes in Gene Expression After NGF Withdrawal

It has been 25 years since Martin *et al.*^10^ reported that RNA and protein synthesis are necessary for neuronal death caused by NGF deprivation and proposed that *de novo* gene expression is required for cell death to occur. This key study contributed to the idea of apoptosis as an active form of cell death and led to the search for proapoptotic genes that are induced in NGF-deprived sympathetic neurons. Early studies based on specific hypotheses identified *cyclin D1*, *c-jun* and *mkp1* (MAP kinase phosphatase 1), among others, as genes upregulated after NGF withdrawal.^63, 64, 65^ c-Jun is a member of the Jun and Fos family of basic/leucine zipper transcription factors, which together with activating transcription factor 2 (ATF2) constitute the transcription factor activator protein-1 (AP-1). c-Jun/c-Fos heterodimers bind to the AP-1 site (5′-TGACTCA-3′) with high affinity, whereas c-Jun/ATF2 heterodimers prefer to bind to ATF sites (5′-TGACGTCA-3′). The *c-jun* mRNA and protein increase in level soon after NGF withdrawal, whereas the other members of the AP-1 family do not change in level.^64, 65^ The microinjection of c-Jun antibodies or expression of a dominant-negative c-Jun mutant or a conditional knockout of the *c-jun* gene in sympathetic neurons protects the cells against NGF withdrawal-induced death^64, 65, 66^ and suggests that the transcriptional induction of AP-1 target genes is important for cell death following NGF deprivation. c-Jun N-terminal phosphorylation also increases after NGF withdrawal.^65, 67, 68, 69^ This phosphorylation increases the transcriptional activity of c-Jun and is mediated by JNKs.^70^

Several other NGF withdrawal-regulated genes that promote neuronal apoptosis were discovered either by looking at the expression of specific genes (*bim*, *p63*, *puma*) or in mRNA differential display experiments (*dp5*, *egln3* (Egl nine homologue 3)).^13, 37, 47, 48, 51, 71^ For each of these genes, the mRNA and protein increases in level after NGF withdrawal and experiments with knockout mice have demonstrated that *bim*, *puma*, *p63* and *egln3* are required for normal NGF withdrawal-induced death.^13, 37, 48, 51, 57, 71, 72, 73^ The BH3-only protein genes *bim* and *dp5* are direct targets of c-Jun,^37, 52, 74, 75^ and the Dp5 and Bim proteins, together with Puma and Bmf, promote MOMP after NGF withdrawal (Figure 3). However, other genes induced after NGF deprivation may be important in other aspects of NGF withdrawal-induced death, for example, the inhibition of protein synthesis and growth or the regulation of specific intracellular signalling pathways. Gene microarray technology has now been used to study the pattern of expression of all known genes in NGF-deprived sympathetic neurons.^53^ Using Affymetrix exon arrays, 415 up- and 813 downregulated genes were identified, including most of the genes previously known to be regulated by NGF withdrawal. One of the known induced genes was *mkp1*, which encodes a mitogen-activated protein kinase (MAPK) phosphatase that can dephosphorylate JNKs. Mkp1 is part of a negative feedback loop induced by the JNK-c-Jun signalling pathway, which inhibits JNK activity and thereby modulates the rate of neuronal death following NGF withdrawal.^14^

The expression of two members of the p53 family, ΔNp73 and TAp63, changes in sympathetic neurons following NGF withdrawal. ΔNp73, an N-terminally truncated isoform of p73, decreases in level after NGF deprivation and has been shown to promote the survival of sympathetic neurons.^76^ ΔNp73 may function by acting as an antagonist of p53 family transactivator proteins or by binding directly to JNKs and inhibiting their activity.^77^ Following NGF withdrawal, TAp63, which is a transcriptional activator closely related to p73, increases in level.^13^ Overexpression of TAp63 induces neuronal apoptosis in the presence of NGF and *p63−/−* sympathetic neurons are resistant to NGF withdrawal-induced death, suggesting that p63 has an important role in developmental neuronal apoptosis.^13^

The use of an MLK inhibitor, CEP-11004, which inhibits the activation of the JNK-c-Jun pathway after NGF withdrawal, has allowed the identification of NGF withdrawal-regulated genes that may be downstream targets of the JNK pathway.^53, 78^ However, the induction of some genes, such as *egln3*, which encodes a prolyl hydroxylase that destabilises hypoxia-inducible factor (HIF), is not affected by CEP-11004.^53^ *Egln3* transcription may be regulated by other transcription factors that are activated after NGF withdrawal, but not regulated by the JNK pathway. Under normal oxygen tensions, the EglN3 protein hydroxylates specific proline residues in HIF-1*α* and HIF-2*α*. The hydroxylated proteins are then bound by the von Hippel–Lindau protein and its associated E3 ubiquitin ligase, resulting in their polyubiquitination and degradation by the proteasome. It has been proposed that EglN3 may promote the death of NGF-deprived neurons in part by suppressing a HIF-2*α*-mediated survival pathway.^79^

## Receptors and Intracellular Signalling Pathways that Regulate Sympathetic Neuron Survival

NGF was the first growth factor to be discovered^80^ and regulates the growth, survival and differentiation of sensory and sympathetic neurons by binding to two types of cell surface receptor: the TrkA tyrosine kinase and the p75NTR, which are often present on the same cell. Binding of NGF to TrkA leads to receptor dimerisation and tyrosine residue phosphorylation of the cytoplasmic tail by adjacent Trk receptors.^81^ When NGF is bound to TrkA, the receptor transmits positive signals that enhance sympathetic neuron growth and survival.^82^ The binding of NGF to TrkA activates the small GTPase Ras, which promotes neuronal survival by activating the PI3K-Akt and Raf-MEK-ERK pathways and also by inhibiting the JNK pathway.^83^ The p75NTR receptor can transmit survival signals with TrkA in response to NGF, and also induces cell death upon binding BDNF (which does not bind to TrkA). Experiments with *p75NTR−/−* knockout mice demonstrated that p75 is required for the NGF withdrawal-induced death of sympathetic neurons and activation of p75NTR using BDNF increases the N-terminal phosphorylation of c-Jun and induces apoptosis.^84^ Subsequent studies showed that p75NTR can activate the JNK pathway specifically through JNK3 in sympathetic neurons.^85^ Recently, it was suggested that TrkA can behave as a dependence receptor and induces the death of sympathetic neurons in the absence of NGF and this TrkA-induced death requires p75NTR.^86^ However, the biochemical mechanism by which TrkA functions as a dependence receptor has not yet been reported.

**Figure 4:**
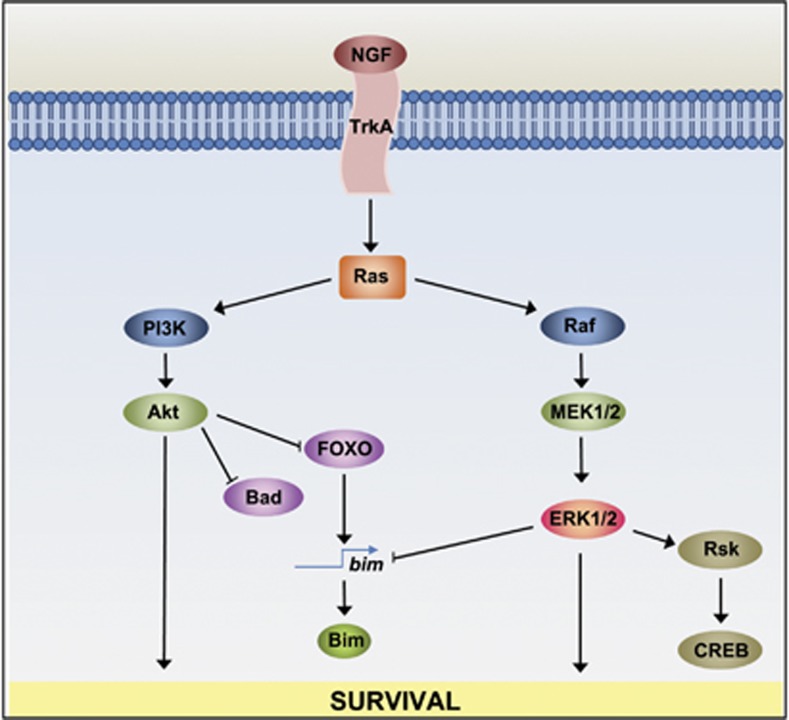
Survival pathways activated by the binding of NGF to TrkA. The binding of NGF to its receptor TrkA can activate the PI3K-Akt signalling pathway, which can inhibit apoptosis and promote cell survival. The binding of NGF to TrkA triggers the activation of the small GTP-binding protein Ras. The subsequent activation of Akt through PI3K can inhibit apoptosis by phosphorylating, and therefore inactivating, proapoptotic proteins such as the BH3-only protein Bad and the transcription factor FOXO. The binding of NGF to its receptor TrkA can also activate the Raf-MEK-ERK signalling pathway. This pathway promotes survival by inhibiting the expression of Bim, and by activating Rsk, which phosphorylates and activates the transcription factor CREB, which can activate the transcription of the *bcl-2* gene

When NGF binds to TrkA, the PI3K-Akt pathway is activated^82^ (Figure 4) and promotes both the survival and growth of sympathetic neurons.^87^ Akt can inhibit apoptosis by phosphorylating and thereby inhibiting the BH3-only protein Bad and the transcription factor forkhead box O3a (FOXO3a). FOXO3a can induce Bim expression when overexpressed and promotes the death of sympathetic neurons in a Bim-dependent manner.^50^ NGF withdrawal causes a rapid decrease in PI3K and Akt activity, which leads to an increase in the amount of FOXO3a in the nucleus, where it induces the transcription of proapoptotic genes such as *bim*. The binding of NGF to TrkA also activates the Raf-MEK-ERK signalling pathway, which can inhibit apoptosis and promote cell survival (Figure 4).^67, 82^ This protein kinase cascade has many effects on neurons, such as an increase in axonal growth in sympathetic neurons after ERK activation.^88^ The Raf-MEK-ERK pathway may suppress apoptosis by phosphorylating and inactivating the Bim protein^89, 90^ and by activating the protein kinase Rsk. Rsk phosphorylates and thereby activates the prosurvival transcription factor CREB^91^ (Figure 4). Furthermore, the Raf-MEK-ERK pathway can reduce the level of *bim* RNA in sympathetic neurons and inhibition of both the PI3K and ERK pathways increases the level of the *bim* RNA to a similar extent as NGF withdrawal.^92^ However, although the ERK pathway clearly regulates the activity of proteins that promote or inhibit apoptosis in sympathetic neurons, such as Bim and CREB, it appears to have a more minor role than the PI3K-Akt pathway in promoting sympathetic neuron survival in the presence of NGF.^82, 92, 93^

**Figure 5:**
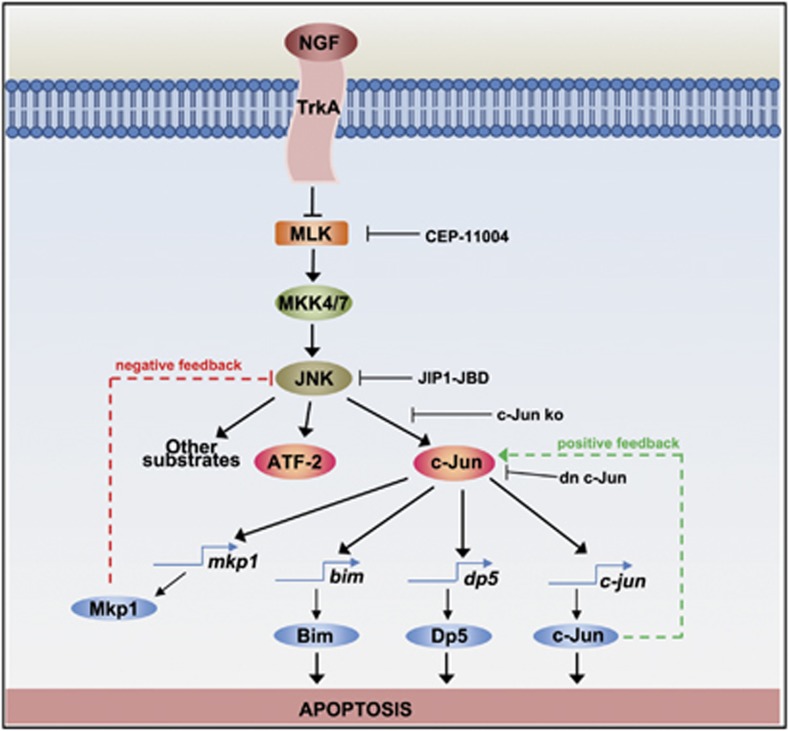
Proapoptotic signalling pathways activated in NGF-deprived sympathetic neurons. When sympathetic neurons are deprived of NGF, the MLK-JNK-c-Jun pathway is activated. MLKs phosphorylate MAP kinase kinases such as MKK4/7, which in turn phosphorylate JNKs. JNK activity increases leading to the phosphorylation of the AP-1 transcription factors c-Jun and ATF2. This increases the ability of c-Jun to activate the transcription of target genes such as *bim*, *dp5* and *c-jun* itself. c-Jun can also bind to AP-1 sites in the promoter of *mkp1*, which encodes a MAPK phosphatase that acts as a negative regulator of the JNK pathway. The MLK inhibitor CEP-11004, which prevents JNK activation, and dominant-negative c-Jun reduce the induction of c-Jun, Bim and Dp5 and block cell death

In sympathetic neurons, NGF withdrawal leads to the activation of the stress-responsive MLK-JNK-c-Jun protein kinase cascade^64, 65, 67, 68, 69, 94, 95^ (Figure 5). NGF withdrawal has been proposed to promote the formation of a complex comprising the multidomain protein, plenty of SH3 domains (POSH), which acts as a scaffold that brings together the small G-protein Rac1, which is an activator of the JNK pathway,^96^ and the other elements of the JNK pathway,^97^ and thereby stimulates the phosphorylation of c-Jun. Recent studies showed that Sh3rf2, a homologue of POSH, acts as an inhibitor of the MLK-JNK pathway.^98^ Following NGF withdrawal, Sh3rf2 levels decrease, which stabilises POSH, activates JNKs and leads to cell death. The JNK-c-Jun dependent transcriptional programme is required for apoptosis induced by NGF deprivation and is initiated by the phosphorylation of the Thr-X-Tyr motif of JNKs by MAP kinase kinase 4/7 (MKK4/7).^37, 64, 65, 66, 70, 99^ Evidence for a role of the MLK-JNK pathway in neuronal death has come from studies using the MLK inhibitor, CEP-1347, and its derivative, CEP-11004,^78, 100^ and from experiments using the JNK-binding domain (JBD) of JNK-interacting protein-1. The JBD is a direct and specific inhibitor of JNKs and expression of the JBD inhibits c-Jun phosphorylation and inhibits NGF withdrawal-induced apoptosis.^101, 102^ JNKs phosphorylate c-Jun at serines 63 and 73 and threonines 91 and 93, which increases the ability of c-Jun to activate the transcription of its target genes,^70^ which include *c-jun* itself, *bim*, *dp5* and *mkp1* in sympathetic neurons (Figure 5).^14, 52, 53, 69^

Another protein kinase-dependent proapoptotic pathway that is activated after NGF withdrawal involves cell-cycle-related proteins. In NGF-deprived sympathetic neurons, activation of the cyclin-dependent kinases (Cdk-4 and Cdk-6) leads to the phosphorylation of retinoblastoma protein (Rb) family members, which causes the dissociation of E2F-Rb (E2 promoter binding factor-Rb) family repressor complexes and consequent derepression of E2F target genes, including *b-myb* and *c-myb*. Small-molecule Cdk inhibitors or dominant-negative forms of Cdk-4 or Cdk-6 protect NGF-deprived sympathetic neurons from death, suggesting that Cdk activation may have a role in the mechanism by which NGF withdrawal triggers neuronal death.^103, 104^ siRNAs that knock down the level of the b-Myb (b-myeloblastosis oncogene) and c-Myb transcription factors protect sympathetic neurons against NGF withdrawal-induced death.^105^ Furthermore, there are two Myb binding sites in the *bim* promoter and mutation of these binding sites prevents the induction of a *bim* promoter – luciferase reporter construct (*bim*-LUC) after NGF withdrawal.^106^

The expression of Bim in sympathetic neurons is tightly regulated at both the transcriptional and translational level.^35^ The *bim* mRNA increases in level after NGF withdrawal, but this, as well as the increase in Bim protein level, can be partially reduced by expressing dominant-negative c-Jun or by using the MLK inhibitor CEP-11004 or by the *Jun*^*AA*^ knock-in mutation in mice, which changes serines 63 and 73 in c-Jun to alanines.^37, 48, 49, 51, 53^ This indicates that c-Jun contributes to the increased expression of Bim after NGF withdrawal, and also suggests that other transcription factors must cooperate with c-Jun to achieve the maximal induction of Bim expression. Two conserved binding sites for FOXO transcription factors were identified in the promoter and first intron of *bim* and these sites are required for the induction of a *bim*-LUC reporter construct after NGF deprivation and FOXO activity is required for normal NGF withdrawal-induced death.^50, 107^ More recently, the identification of an inverted CCAAT box (ICB) in the *bim* promoter revealed that the trimeric transcription factor NF-Y (nuclear transcription factor Y) binds to the *bim* ICB and both the ICB and NF-Y activity are essential for the induction of the *bim*-LUC reporter after NGF withdrawal.^108^ Furthermore, it was shown that NF-Y and FOXO3a interact with the coactivators CBP and p300 after NGF deprivation and that CBP/p300 activity is required for the activation of the *bim* promoter. CBP/p300 may integrate the different signalling pathways that cooperatively activate the *bim* promoter after NGF withdrawal through the DNA binding transcription factors c-Jun, FOXO3a and c-Myb.^50, 74, 107, 108^

## Other Signals that Induce Apoptosis in Sympathetic Neurons

Sympathetic neurons have been an important model for studying NGF-regulated developmental neuronal apoptosis. However, sympathetic neurons have also been very useful for studying how other signals induce neuronal death, including DNA-damaging agents, glial cell line-derived neurotrophic factor (GDNF) withdrawal, ER stress and mutant polyQ-expanded Huntingtin (Htt) protein. In experiments using the DNA-damaging agent cytosine arabinoside (araC), apoptosis is induced in sympathetic neurons via a p53-dependent JNK-independent mechanism. In this context, the inhibition of the ERK pathway increases the rate of apoptosis induced by araC in the presence of NGF, suggesting a protective role for the ERK pathway.^109^ GDNF is a neurotrophic factor that can promote the survival of ∼34% of SCG neurons isolated from 1- or 2-day-old rats.^110^ When these cells are deprived of GDNF, a novel non-mitochondrial caspase-dependent death pathway is activated in the neurons, which does not involve cytochrome *c* release, Bax, caspase-3 or caspase-9.^110^ Sympathetic neurons have also been used to identify the pathway of apoptosis triggered by ER stress, which is often associated with various pathological conditions. ER stress induces a neuronal apoptotic pathway that upregulates the BH3-only genes *dp5* and *puma* and commits neurons to die before cytochrome *c* release, which requires Bax activation and JNK signalling. It also highlights the importance of the apoptosome as the non-redundant caspase activation pathway to execute neuronal apoptosis in response to ER stress.^111^ Finally, the effect of a polyQ-expanded Htt protein, which causes Huntington's disease, has been studied in sympathetic neurons. In the presence of NGF, expression of an expanded polyQ Htt aggregating protein causes sympathetic neurons to die, but only slowly, because they express HSP70, which diverts apoptosis into slow necrosis.^112^

## Future Directions

Our current state of knowledge of the mechanisms of apoptosis in sympathetic neurons will allow researchers in the field to investigate a number of interesting questions in the future. Although much has been learned about the role of Bcl-2 family members in this system, many important questions remain to be answered. For example, what is the consequence of knocking out both *bim* and *puma* in sympathetic neurons? And, how is the expression of these genes regulated by NGF withdrawal? Some work has been carried out on the transcriptional control of *bim*, but relatively little is known about *puma* in sympathetic neurons. In other cell types, *puma* is a direct target of p53^113, 114, 115, 116^ and also a target of FOXO3a.^117^ When sympathetic neurons are treated with araC, p53 is activated and the expression of Puma increases, and the resulting araC-induced apoptosis depends on *p53* and *puma*.^57^ Therefore, *puma* might be directly regulated by p53 family members or FOXO transcription factors after NGF withdrawal. Exon microarray experiments have demonstrated that many other genes are induced after NGF withdrawal.^53^ Like *c-jun*, *mkp1*, *dp5* and *bim*, some of these are likely to be downstream targets of the JNK-c-Jun pathway or FOXO transcription factors and it will be interesting to investigate their function in sympathetic neurons and study how their expression is regulated.

Sympathetic neurons depend on target-derived NGF for survival during late embryonic and early postnatal development,^5^ but become independent of NGF at later stages of development.^118^ This switch can be reproduced *in vitro* by culturing P1 sympathetic neurons for 28 days in NGF-containing medium. These 28 DIV neurons do not die when deprived of NGF, even though the JNK-c-Jun pathway is activated in these cells. The molecular basis for this phenomenon was investigated by Wright *et al.*,^119^ who observed that Apaf-1 expression is greatly reduced in 28 DIV neurons and P28 superior cervical ganglia owing to alterations in the chromatin structure of the *apaf-1* promoter.^119^ In a more recent study of the changes that occur during the postnatal development of SCG neurons, Kole *et al.*^54^ reported that the microRNAs (miRs) *MiR-29a*, *-29b* and *-29c* greatly increase in level in SCGs from P13 onwards.^54^ They found that increased expression of *MiR-29* may contribute to the resistance of P28 sympathetic neurons to NGF withdrawal-induced death because *MiR-29* binds to the 3′-UTR regions of the mRNAs that encode Dp5, Bim, Puma and Bmf and inhibits their expression^54^ (Figure 3). It will be interesting to study SCG neurons in *MiR-29−/−* knockout mice and to determine how the expression of *MiR-29a*, *-29b* and *-29c* is regulated during the postnatal development of SCG neurons.^118^

Another important future research direction will be to understand more precisely how the signalling pathways that are regulated by NGF withdrawal are activated or inhibited by the removal of NGF. TrkA has been reported to function as a dependence receptor and induces cell death in the absence of NGF.^86^ It will be interesting to work out how this proapoptotic function of TrkA is linked to the activation of the MLK-JNK-c-Jun pathway. At the same time, NGF withdrawal inactivates the prosurvival PI3K-Akt pathway.^82^ This inactivation appears to be mediated in part by the Trib3 protein, which increases in level after NGF withdrawal.^120^ Trib3 inhibits Akt activity and thereby activates FOXO3a, which in turn binds to and activates the *trib3* promoter.^120^ This constitutes a feedforward loop and it will be important to identify other molecules that function in regulatory loops that activate or inhibit the MLK-JNK-c-Jun and PI3K-Akt pathways in sympathetic neurons.

A recent study investigated the role of components of the mitochondrial pathway in axon degeneration induced by local NGF deprivation *in vitro*.^121^ In these experiments, the sympathetic neurons were cultured in microfluidic chambers so that the cell bodies and distal part of the axons were in separate compartments. Removal of NGF from the distal axons induced axon degeneration, but did not trigger apoptosis in the cell bodies, which were protected by XIAP. This axon degeneration required Bax, caspase-9, caspase-3, caspase-6 but not Apaf-1.^121^ This suggests that local NGF deprivation induces an alternative caspase activation pathway that is independent of the conventional apoptosome and this will certainly be an interesting area of research in the future.

Finally, some of the molecular mechanisms that were discovered using the sympathetic neuron model have also been studied in other models of developmental neuronal apoptosis and in cell culture and *in vivo* models of neurodegeneration. For example, the JNK-c-Jun pathway was first reported to promote cell death in sympathetic neurons and differentiated PC12 cells following NGF withdrawal.^64, 65, 67^ It was then found to have a proapoptotic role in some animal models of neuronal injury or neurodegeneration including the kainic acid-induced excitotoxic death of hippocampal neurons,^122, 123^ cerebral ischaemia-induced cortical neuron death,^124^ the MPTP-induced death of dopaminergic neurons, which is a model for Parkinson's disease^125^ and in the optic nerve crush-induced death of retinal ganglion cells.^126^ It will be interesting to see which other features of NGF withdrawal-induced death are important for neuronal apoptosis in other regions of the developing and adult nervous system. In conclusion, it is likely that the sympathetic neuron model will continue to be much studied in the future and will provide further important insights that increase our understanding of the mechanisms of neuronal apoptosis during normal neural development and following injuries to the nervous system.

## Abbreviations

Akt: murine thymoma viral (v-akt) oncogene homologue
AP-1: activator protein-1
Apaf-1: apoptotic protease-activating factor-1
AraC: cytosine arabinoside
ATF2: activating transcription factor 2
Bad: Bcl-2-associated agonist of cell death
Bak: Bcl-2-antagonist/killer
Bax: Bcl-2-associated x protein
Bcl-2: B-cell CLL/lymphoma 2
Bid: BH3-interacting domain death agonist
Bim: Bcl-2-interacting mediator of cell death
Bmf: Bcl-2-modifying factor
Cdk: cyclin-dependent kinase
CGN: cerebellar granule neuron
c-Jun: Jun proto-oncogene
CNS: central nervous system
Dp5: neuronal death protein Dp5
E2F: E2 promoter-binding factor
Egln3: Egl nine homologue 3
ERK: extracellular signal-regulated kinase
FOXO3: forkhead box O3
GDNF: glial cell line-derived neurotrophic factor
HIF: hypoxia-inducible factor
Htt: Huntingtin protein
JNK: c-Jun N-terminal kinase
MAPK: mitogen-activated protein kinase
MEK: MAPK/extracellular signal-regulated kinase kinase
Mkp1: MAP kinase phosphatase 1
miR: microRNA
MKK4: MAP kinase kinase 4
MKK7: MAP kinase kinase 7
MLK: mixed lineage kinase
MOMP: mitochondrial outer membrane permeabilisation
Myb: myeloblastosis oncogene
NF-Y: nuclear transcription factor Y
NGF: nerve growth factor
P1: postnatal day 1
p75NTR: p75 neurotrophin receptor
PI3K: phosphatidylinositol 3-kinase
PNS: peripheral nervous system
POSH: plenty of SH3 domains
Puma: p53 upregulated modulator of apoptosis
RAIDD: RIP-associated ICH-1/CAD-3 homologous protein with a death domain
Rb: retinoblastoma protein
SCG: superior cervical ganglion
TUNEL: terminal deoxynucleotidyl transferase dUTP nick-end labelling
XIAP: X-linked inhibitor of apoptosis protein
